# Save the tree of life or get lost in the woods

**DOI:** 10.1186/1745-6150-5-44

**Published:** 2010-07-01

**Authors:** Ruben E Valas, Philip E Bourne

**Affiliations:** 1 Bioinformatics Program, University of California, San Diego, 9500 Gilman Drive, La Jolla, CA 92093, USA; 2 Skaggs School of Pharmacy and Pharmaceutical Sciences, University of California, San Diego, 9500 Gilman Drive, La Jolla, CA 92093, USA

## Abstract

**Background:**

The wealth of prokaryotic genomic data available has revealed that the histories of many genes are inconsistent, leading some to question the value of the tree of life hypothesis. It has been argued that a tree-like representation requires suppressing too much information, and that a more pluralistic approach is necessary for understanding prokaryotic evolution. We argue that trees may still be a useful representation for evolutionary histories in light of new data.

**Results:**

Genomic data alone can be highly misleading when trying to resolve the tree of life. We present evidence from protein abundance data sets that genomic conservation greatly underestimates functional conservation. Function follows more of a tree-like structure than genetic material, even in the presence of horizontal transfer. We argue that the tree of cells must be incorporated into any new synthesis in order to place horizontal transfers into their proper selective context. We also discuss the role data sources other than primary sequence can play in resolving the tree of cells.

**Conclusions:**

The tree of life is alive, but not well. Construction of the tree of cells has been viewed as the end goal of the study of evolution, where in reality we need to consider it more of a starting point. We propose a duality where we must consider variation of genetic material in terms of networks and selection of cellular function in terms of trees. Otherwise one gets lost in the woods of neutral evolution.

**Reviewers:**

This article was reviewed by Dr. Eric Bapteste, Dr. Arcady Mushegian, and Dr. Celine Brochier.

## Background

The pendulum of scientific opinion often swings back and forth in the light of new data and hypotheses. 150 years ago Darwin's observations pushed opinion towards believing the universal tree of life (TOL) existed for the first time [1]. This view was pushed to an extreme 30 years ago as Woese pioneered the use of sequence data to build universal trees [2]. But the pendulum has begun to swing back the other way in the past decade, as a wealth of prokaryotic genomic data has demonstrated a higher than expected frequency of horizontal gene transfer (HGT).

Ford Doolittle and Eric Bapteste's arguments against the TOL hypothesis are quite compelling [3], and this view seems to be gaining support [4]. These authors argue that HGT is so rampant that tree-like representations of prokaryotic species contain too little information to capture evolutionary histories. Their work questions whether the metaphor of the TOL is inspired from a historical bias from the taxonomy of eukaryotes, and therefore should not be applied to prokaryotes. This is an important and worthwhile question to ask. Resolving the eukaryotic tree is a distinct problem because there is much less horizontal transfer and a much better preserved fossil record. The conclusion of Doolittle and Bapteste is not so much that the inability to build the tree is the problem, rather it is forcing the data into a tree that needs to be questioned, and in a pluralistic framework, avoided, since this model does not allow a precise description of the evolutionary processes.

The TOL and tree of cells (TOC) should be one and the same. However, the meaning of the former has become the trees we can build, and the latter has become the hypothetical tree we cannot build. This difference was recently discussed in [5]. The reason the TOC is truly a tree is simple and has been stated by many before us. Every extant cell on this planet is the daughter of a cell that came before it [6]. Prokaryotic cells divide by binary fission. Therefore there must truly be a TOC in the prokaryotic superkingdoms. Nobody seems to dispute this. If every daughter cell's membrane kept track of who its parent was, reconstructing the evolution of cell divisions would be a trivial task. But since there is no selective pressure for cells to do that, so we are left with a more difficult task.

Since the membranes do not keep track of heredity we chose a different representation of ancestry, the genome. The genetic material of the cell does keep track of its parents in some sense as there is selective pressure to ensure fidelity of replication. All of the issues the community is currently having with the TOL hypothesis stem from the simple fact that genomes are not a perfect representation of membrane history. Membrane heredity is a tree-like structure, but all of the recent work on the pervasiveness of HGT has shown that genome heredity is often more of a network than a tree. We are beginning to have enough technology to reconstruct genomic evolution, but we are only beginning to realize how vastly different that is from cellular evolution. However, even genomic evolution makes little sense without the light of cellular evolution.

Ernest Rutherford said, "Physics is the only real science. The rest are just stamp collecting". Some biologists have taken this as a challenge to create universal laws in biology on par with those in physics. This is a noble endeavor, and has produced many interesting results, but the goal should to be to collect stamps in a way that is justified by the laws. The promise of the TOL did just that. It was a collection of every living thing as well as the laws that organized that collection.

Instead of a consensus TOL emerging from the vast amount of genomic data available, the community was faced with the disappointment that very few genes are universally conserved. The universal sequence tree created from 31 concatenated proteins [7] has been criticized as "the tree of 1%" because the average prokaryote genome has about 3000 genes [8]. They argue that even if this gene set did produce a reliable tree it would only reflect a small portion of evolution, since this is such a tiny portion of the genome. The assumption that genomic histories were congruent with cellular histories hid the fact that much of the collection could not be explained by the TOL hypothesis. The community was lost in the woods without knowing it under tree monism.

We worry that the sound arguments against the TOL hypothesis will shift focus away from evolutionary histories. For instance Dagan *et al. *[9] have quantified rates of horizontal transfer for every gene family. This is a tour de force of quantifying a law in biology. However, they do not give examples of the rates for any specific family, or cite any example they found where a horizontal transfer played a role in speciation. They also say their results are independent of the vertical tree used as input, which we find worrying. The overall rates of HGT may not change, but we assume the rates for each family almost certainly would. Not worrying about that difference is getting lost in the woods to us because the real history of the stamp collection is lost in search of a concise law.

Here we argue for the need to be cautious about how far away from the TOL hypothesis we swing, as novel sources of data already bring into question the conclusions supported by genomics. The arguments against the TOL are centered on the idea that the modern synthesis of biology from 50 years ago was too eukaryote-centric. We hope to offer a perspective that will spare this current synthesis from being labeled too genome-centric 50 years from now. We are not arguing for tree monism. Instead we are attempting to demonstrate that the TOC becomes even more important under a pluralistic approach. Not all genes contribute equally to the cell, and we will demonstrate that vertical inheritance of function has a more pronounced signal than vertical inheritance of genetic material. Using only universal sequences to attempt to resolve the TOC is a narrowing strategy, and we will discuss alternative sources of data that may still shed light on this problem.

## Results and Discussion

### The Great Tree of Cellular Function

The attempts at resolving the TOL using universal sequences only represent a small part of the history of genomes, but what portion of the history of cells does it represent? Genomic methods represent all genes equally. Subsequently, a gene that is only expressed under specific conditions can be just as useful as a housekeeping gene for building a species phylogeny if both are present across the same set of genomes. If we created a concatenated protein sequence, both proteins would be counted as equals proportional to their length despite vast differences in their actual abundance as proteins in the cell. Here lies the fundamental shortcoming of genomics; confusing the genome and the cell. If we wish to measure a gene's contribution to the cell there are many different metrics: essentiality, abundance of proteins, number of transcripts, and portion of total weight are just a few. Any of these will give dramatically different proportions than simply counting the copy number within the genome.

The abundance of many proteins present in *Escherichia coli's *cytoplasm has recently been calculated experimentally [10] as well as for the entire cell of *Leptospira interrogans *[11]. For the first time data are available to measure what portion of a prokaryotic cell each protein comprises. All of these numbers should be taken with a grain of salt due to experimental noise, but the trend is clear; the core proteins make up a larger portion of the cell than the genome. The data used to calculate these values are available as Additional files 1 and 2. We argue abundance is a good proxy for evolutionary importance because there is a correlation between the abundance of a protein and the energy a cell invests into producing it. It has been demonstrated that highly expressed proteins have been optimized to use less energetically costly amino acids [12], and that highly abundant proteins are shorter on average [10]. The abundant proteins justify the use of a large portion of the cell's energy despite these optimizations, so they must be important. Proteins perform most of the functions in the cell. Comparing how many of the same functions two cells are doing at the same time is a good measure of similarity. The downside of abundance is it is dynamic during a single generation, while genomes are static. This makes direct comparison more difficult, but it still gives insight into the evolution of genomes. Our point is not that this data magically fixes all problems with the TOL hypothesis, but rather that many important details are left unresolved in our understanding of the big picture that still may come into focus.

Let us consider the so called "tree of 1%". The authors list 36 genes that are universal but claim that only 31 have not been horizontally transferred [7], although later analysis claims the number is actually 22 [13]. However, there are arguments that a TOL is still meaningful despite a large incongruence between individual gene trees [14], but a detailed argument against that view is presented in [4]. 34 of these genes are present in the *E. coli *data set that measures abundance for 1103 proteins. For this argument let us consider the universal set because in this case the HGTs appear to be displacements of genes that were already present. That is to say the function of these genes was vertically inherited despite HGTs of the genetic material. This brings up the point that there are two distinct forms of HGT that we need to consider that are currently not distinguished enough; functional innovations (relative to the recipient genome) and displacements.

For example, the histories of the 20 aminoacyl-tRNA synthetases (AARSs) contain many inconsistencies, the result of HGTs [15]. However, it still appears that most of these enzymes date back to the last universal common ancestor (LUCA), with the possible exceptions of AsnRS and GlnRS. HGT makes many of these proteins unusable for reconstructing a universal TOC as their sequence trees would be inconsistent with the cellular history. However, the HGTs of this family would displace a copy of the gene that was already present. In that sense there is no functional innovation caused by the transfer. Even though the genetic material was inherited horizontally the functional content was still transmitted vertically, and would still be consistent with the TOC. We argue events like this are far more deleterious to tree reconstruction algorithms than they are to the recipient cells. Current methods for estimating HGT rely on measuring inconsistencies between sequence trees or looking for unusual compositional features [16], so there is no way for them to distinguish between innovations and displacements. We must also consider the role functional redundancy plays in prokaryotes. There are nearly two hundred known cases of non homologues enzymes performing analogous reactions [17]. HGT of such enzymes should be relatively easy since they can plug into existing metabolic pathways. Therefore any current measure of the vertical inheritance of genetic material is a lower bound on functional vertical inheritance. If one wishes to measure the size of the vertical component of evolution it must be done in terms of function as well as genetic material.

The universal genes are about 3% of the E. coli dataset if we count all genes equally. However, it we count proteins by abundance, the 34 from the universal core make up 6.6% of the data set (Table 1). That would double the thickness of the vertical component! A tree of 2% may not seem dramatically better than a tree of 1% but the point is that the universal proteins make up a larger portion of the cell than the genome. This gets quite dramatic when one takes into consideration that 84.5% of the abundance in this dataset is made up of ribosomal protein L33. L33 is universal across the Bacteria, but absent in the Archaea. Although it is not universal it still must be very ancient. The fact that a large portion of the cytosolic proteome of an extant cell would be present in the cytosol of LUCA truly speaks to the fact that vertical inheritance can be a major force along long evolutionary time scales. This is consistent with previous work that showed older genes are expressed at higher levels than younger genes [18]. However, the authors did not quantify the contribution of the universal proteins in this manner.

**Table 1:** Coverage in terms of cellular versus genomic abundance in *E. coli*'s cytosol.

Gene Set	Protein Abundance Coverage	Genomic Abundance Coverage
Universal 34	6.57%	3.08%

Core *Enterobacteriaceae*	99.96%	79.22%

All Enterobacteriaceae	99.80%	61.34%

Non-Ribosomal Core Enterobacteriaceae	85.14%	78.10%

Non-ribosomal All Enterobacteriaceae	81.70%	59.33%

The protein abundance from *L. interrogans *[11] provides us an opportunity to test the trends we see in *E. coli*. This data set was not dominated by a single protein like L33 in *E. coli*. The 36 universal proteins make up only about 1% of this genome, but 5.4% of the entire cell's abundance (Table 2). A tree of 5% starts to sound significant! This shows the vertical component of the TOL is five times thicker than genomics alone would lead us to believe. Again, regardless of the nature of LUCA, extant cells still have a significant amount of function in common with her.

**Table 2:** Coverage in terms of cellular versus genomic abundance in a *L. interrogans cell*.

Gene Set	Protein Abundance coverage	Protein Abundance Coverage
Universal 36	5.09%	0.96%

Spirochete Core	30.99%	11.28%

The TOL hypothesis has also been challenged on the grounds that currently defined taxa may have a very small genomic core (the intersection of their gene sets) and very large pan genomes (the union of their gene sets) [3,4]. We analyzed the genomic cores' abundances to explore their contribution to the cell. A subset of the proteins in any Enterobacteriaceae genome are conserved in enough genomes to be considered part of their genomic core, and even fewer are conserved across all nine genomes studied in [19]. This is a very diverse set of bacteria ranging from endosymbionts to free-living species. In each case the coverage in terms of genes is much smaller than the coverage in terms of protein abundance (Table 1). The most dramatic example is that the genes conserved in all nine genomes only account for about 61% of the genes in this dataset, but they account for 99.8% of the protein abundance! Since the ribosomal proteins are so dominant in the data set, we repeated the same measures excluding ribosomal proteins as was done in the initial study. The results are not as impressive, but in each case the core proteins are up to 20% more abundant in the cell than they are in the genome. Measuring the similarity of species based on shared gene content greatly underestimates their functional conservation.

We also considered a set of genes conserved across four Spirochete genomes [20] that includes both obligate and non-obligate parasites. 412 proteins that were conserved across four Spirochete species were mapped to the abundance data set. This accounts for only 11.3% of the *L. interrogans *genome, but makes up 31% of the cell when counted by abundance (Table 2). There is stronger conservation between these species than genomic data implies. Again, even though these species have vastly different lifestyles a large cellular core has remained conserved between them.

**Figure 1:**
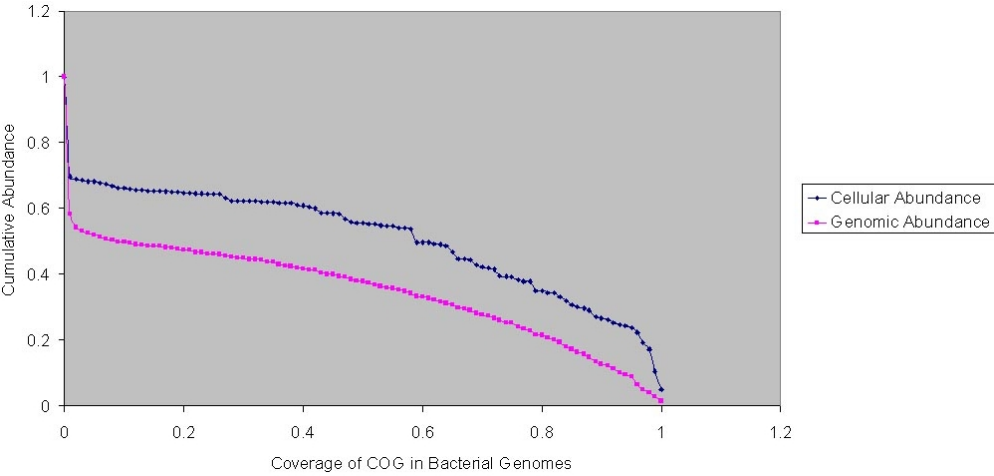
**Genomic vs Cellular Abundance in *L. interrogans***. Each COG's presence in bacterial genomes was plotted against the cumulative abundance of all COGs that are present in at least that many genomes. The conserved genomic core is always an underestimate of the conserved cellular core, in some places by as much as 20%.

We assigned COGs (clusters of orthologous genes) to every gene in the *L. interrogans *dataset using the STRING database [21]. This allowed us to compare the relative age of a COG (the percentage of bacterial genomes that have a particular COG) to the genomic or cellular portion of this dataset that COG composes. A cumulative plot of genomic and cellular abundance reveals that at every level genomic abundance underestimates cellular abundance (Figure 1). In some cases the difference can be as high as 20%. A similar plot for the *E. coli *dataset was not informative because the dominating feature is ribosomal protein L33 (data not shown).

We mapped the tree inconsistency scores (ISs) for all trees from the forest of life in [22] to these datasets to test our proposal that abundance is a barrier to HGT. IS is defined as the frequency the splits in a specific tree are found in all single gene phylogenies in the forest of life. Therefore it is an estimate of the horizontal transfer rate for each family. The authors of that work found that ISs have a bimodal distribution, with many families having very low IS (below .6 of the average IS) or very high IS (above 1.4 of the average IS). They noticed many ribosomal proteins had very low IS and proposed this is due to their numerous physical interactions. If highly abundant proteins are less likely to be transferred their trees should not have high ISs. However, there may still be proteins that are not abundant with very low ISs for reasons besides abundance, so we do not think a comparison of average ISs is informative. Instead we counted how many high ISs were found in the 100 most abundant proteins that had an IS. We repeated this measure excluding ribosomal proteins to ensure this result is due to abundance and not just physical constraints on protein complexes. P-values were estimated by taking 10,000 permutations of 100 ISs taken out of all possible ISs. In each case they were significantly less high ISs among the most abundant proteins than would be expected by chance (Table 3). Abundance does not mean it is impossible for a transfer to occur, but it certainly seems to limit it. Even though the backbone of vertical inheritance may appear to be dwarfed by HGT on the genomic scale, it is clear that vertical inheritance plays a larger role in the cell than the genome.

**Table 3:** Inconsistency scores (ISs) for the most abundant proteins in each data set.

	Proteins with high IS	P-value
100 most abundant proteins with IS in *L. interrogans*	7	.0067

100 most abundant non-ribosomal proteins with IS in *L. interrogans*	9	.033

100 most abundant non-ribosomal proteins with IS in *E. coli*	6	.0013

100 most abundant non-ribosomal proteins with IS in *E. coli*	9	.033

The most abundant proteins have fewer high ISs which implies abundance is a barrier to HGT.

One of the major criticisms of tree monism is the arbitrary methods used to deal with incongruent data and ambiguities in a single tree of life model [4]. Bapteste *et al. *cite the ambiguous placement of *Aquifex aeolicus *in a supertree as an example of conflicting signals; sometimes they are placed near the Proteobacteria and sometimes near *Thermotoga maritima *[23]. We propose that abundance could be used to consider the relative weights of two incongruent signals such as these. We predict the vertical signal would become clear if we could measure the abundance of proteins shared between *A. aeolicus, T. maritima*, and the Proteobacteria. It is not just the number of genes that are shared that matters; one needs to consider what their contribution to the cell is.

### Placing sequence trees in their cellular context

So far we have argued the large role vertical inheritance of function has played in evolution is larger than the network of genomes implies. But how can we proceed forward if that tree is not the one reconstructed by gene sequence trees? We must first discuss the very reasons the TOL metaphor is appealing in the first place. Trees are the natural representation of replicating processes [24]. As discussed in [4] the tree representation has three strengths: 1) it provides a hierarchy for classification; 2) shared traits are implied by that hierarchy; and 3) ancestral traits are inferred from the branch order of the tree. We believe the strength of the hierarchy rests on the last two points so we will address those first.

The branching order of a true species tree can be read like a timeline. It implies the ancestral state for each group, as well as the transition that defined the groupings. Without a timeline much of evolution becomes gibberish. There are many transitions that can be polarized. The biggest transition in evolution is between the prokaryotes and eukaryotes. It is truly impossible to represent endosymbiosis in a tree representation, but an undirected network would just say that the eukaryotes are related to both the archaea and bacteria. However, it is not just the relationships we need to know to understand this event; the key point is the directionality of the transition. The eukaryotes came from the prokaryotes. That is to say there was a time where prokaryotes existed but eukaryotes did not. Trees have many shortcomings for representing prokaryotic evolution, but any data structure that lacks temporality is even worse.

Since there is no real branch order on any of the current universal sequence trees or networks, some take this as evidence that the origin of all the major prokaryotic taxa are contemporaneous (the condensed cladogenesis model) [25] or are the result of intense periods of HGT (the biological big bang model) [26]. If the major events in prokaryotic evolution all happen near the root of the tree, then it might be really be impossible to reconstruct life's history. However, there are no fossils that unambiguously mark the origin of any major prokaryotic taxa (reviewed in [27]). Therefore the justification for both of these models comes from a lack of resolution in both the fossil and sequence data. It is critical we make a distinction between the big picture of evolutionary history being hard to resolve and it not existing.

We argue against the expectation that every major prokaryotic taxa showed up at the same moment in evolutionary history. Not all innovations are possible immediately in evolution; they need the right push in selective pressure from the environment. The most important examples are processes that require an oxygenic atmosphere such as sterol synthesis [28]. Oxygen dependent metabolism could not thrive before the great oxidation event that occurred about 2.3 billion years ago [29]. This can be used to constrain the ages of several branches of the TOL [30].

This begs the question of whether the major prokaryotic taxa are contemporaneous in origin or not. Despite the disagreement between the current macrophylogenies [31-33], they imply the major prokaryotic taxa did not appear at the same time. The disagreement between these phylogenies is not in terms of how to define the major taxa but rather in the proper way to polarize the data, especially the indels (insertion deletions) which we have discussed [34]. However, the distribution of these traits themselves implies specific taxa evolved before another, regardless of the direction of each polarization. For example, there is a large insert in HSP70 (heat shock protein 70) that is present across the Gram-negative bacteria, but absent in the Gram-positives. There is no reason to assume the insertion deletion event occurred early in evolution. That event would be contemporaneous to the change in membrane structure. There must be a branch order between the Gram-positives and Gram-negatives; even if it is not resolved in sequence trees. One would be very hard pressed to draw a detailed scenario of transfers that explains the distribution of fixed indels better than a more timeline like structure. There are numerous similar divides that can be drawn across the prokaryotes. Relatively stable traits like these must used as guides to reconstruct the TOC. If the origins of the major clades really are very close to contemporaneous we should not expect it to be possible to reconstruct a macrophylogeny at all using such traits. Despite their disagreement, we take the macrophylogenies as evidence that the origins of major prokaryotic taxa could not all be contemporaneous. If the clades arose out of intense periods of HGT the indels and other data points should have largely independent histories. Instead many indels appear on the same points on the tree, which act to independently verify each other.

Why would there be more signal left in the rare events instead of the sequence alone? We propose an alternative model where cladogenesis is primarily caused by revolutions in the "abundome." Some proteins that were highly abundant in LUCA may be entirely absent in an extant cell. This would be a result of major events that led to dramatic changes in gene expression of even the most conserved genes. Such a change in the abundome could make some HGTs more deleterious since the protein would be plugged into a modified core. It is possible these events could actually be periods of reduced HGT. Abundance data may make it possible to quantify what Simpson coined "quantum evolution" when referring to the metazoan fossil record [35], the idea that changes in one part of an organism can trigger a domino effect of rapid evolution, on a molecular level in prokaryotes. There is an inverse relationship between population size and evolutionary rate [36]. Initially members of a novel niche could evolve rapidly. It is also possible that could lead to a population size that is large enough to cause extreme purifying selection, essentially freezing ribosomal sequences. It seems impossible to predict the effects cladogenesis will have on sequences without taking into account other sources of data. This view that revolutions in abundances play a role in cladogenesis is supported by the observation that there are major changes in gene regulation between the major prokaryotic taxa [37]. Since gene expression is a major driver of evolutionary rates [38], there is no reason to expect protein sequence to be well behaved across these events even if cellular population size remained constant. That, taken with the new observation that evolutionary rates vary greatly between prokaryotic groups [39] implies our null hypothesis should be that sequence trees would not resolve the branch order of the major prokaryotic groups even in the complete absence of HGT. Therefore we are in complete agreement that a tree created by concatenating protein sequences together is not the TOL or the TOC. But we do not take that as evidence the TOC does not exist, is not resolvable, or is not useful as a concept for understanding prokaryotic evolution. It just means the community needs to move beyond primary sequence analysis.

It will be possible to look at the coevolution between the cellular and genomic cores as abundance data becomes available from more species. This will allow us to divide extant species into groups that have maintained a cellular backbone. Evolution within these groups should be well suited for study using sequence since there will be fewer confounding factors. This view is supported by the growing list of prokaryotic clades that form well defined sequence trees discussed in [5]. If we can identify the innovation between groups that leads to the differences in abundances it may be possible to polarize these transitions in the manner pioneered in [31]. This approach creates a timeline that is appropriate for classification purposes, and thus we approach the advantages of the TOL while reconstructing the TOC. Of course there will be traits that do not fit that timeline and we must consider them in a pluralistic fashion. However, the timeline will allow us to polarize many of the HGTs and place true innovations in their proper context. Current sequence-based methods could be made much less arbitrary by comparing them against these other lines of data. Combining the TOC with genomic histories would capture all the positive aspects of the TOL hypothesis, while accommodating HGT.

It takes a universal sequence that has not been horizontally transferred and has evolved at a steady rate to build a universal tree. As discussed above that does not leave us with very much data. It takes two widely distributed paralogous proteins to polarize an indel [40], which leaves us with even less. Therefore we are very interested in non-ubiquitous traits that may be useful in resolving the branch order of the prokaryotic taxa. We have found protein structure to be a highly useful tool for studying evolution, but hopefully there are others as well. A transition within quaternary structure only requires a protein to be universal within a taxon of interest. We have presented two transitions in quaternary structure that exclude the root from the Archaea: Anbu evolving into the 20s proteasome [41] and PyrD 1A evolving into PyrD 1B [34]. Neither of these proteins is in Ciccarelli *et al*.'s dataset because they are not universal. But they are derived structures that are universal enough in the Archaea to provide compelling independent arguments that exclude the root of the TOL from within the Archaea. Even if the proteasome sequences have been horizontally transferred all over the Archaea it does not take away from the fact all Archaea have a proteasome (those would be horizontal displacements). Therefore a protein might be useful for resolving a branch order in the tree even if there is major incongruence between the cellular and genetic history. It is currently possible to predict the tertiary structure for about half of a prokaryotic proteome [42]. However, quaternary interactions are not being predicted from sequence in the same way. This gives us hope that there are still untapped sources of data that might resolve the branch order of the major prokaryotic taxa.

## Conclusion

We must keep in mind the humor of calling the central metaphor for evolution "the tree of life". The phrase first appears in Genesis 2:9:

And the LORD God made all kinds of trees grow out of the ground--trees that were pleasing to the eye and good for food. In the middle of the garden were the tree of life and the tree of the knowledge of good and evil.

There is irony in using the name of a tree central to the creation story to argue against that very myth. Therefore we doubt that any phrase will ever pack as much punch as the "tree of life", even if the pattern of common descent is more of a web. It is very important that the community stops labeling any tree derived from a single data source the TOL. The recent attempts to resolve the TOC using primary sequence should be labeled "universal sequence trees", a name that is grounded in the limitations of the data. The title TOC should be reserved for branch orders that are supported by several lines of independent evidence, and the TOL should be the synthesis of those branch orders and horizontal process.

Perhaps the most important line of reasoning that the TOL exists is the fact that HGT is so rampant. Why is HGT possible at all? The answer is obviously common descent. If it was not for common descent the genetic code would not be universal, and most HGTs would not even be translatable in their new host. Many biological parts are interchangeable because they have evolved in conjunction with the same systems. Therefore, we argue the very reason the TOC is so hard to reconstruct is because it exists!!

It has now become clear that many expectations about prokaryotic evolution were based too heavily on observations of eukaryotes. There is truly a fundamental divide in the way these two groups use the communal gene pool as a genetic memory [43]. However if we give up on the eukaryotes as a model, it is not clear what our expectation of prokaryotes should be. It does not make sense to us to criticize a tree as "the tree of 1%" without providing a justified cutoff of n% that would be enough for the vertical component of genomic evolution to be meaningful. We think the TOL crisis would be worse if it was the "tree of 99%", as it would be quite difficult to explain the phenotypic differences between humans and *E. coli*. It is remarkable any genes are conserved since LUCA, and therefore the TOL still rings true to us.

Likewise, it is not clear what level of genome conservation between strains of prokaryotes would be satisfying enough to consider them evolving in a tree-like manner. It is true that two strains of the same species may have relatively few genes in common, but we have argued above this is probably an exaggeration of functional distance. Abundance data from different strains under similar growth condition will shed light on their true functional differences. We predict this gap will be much smaller than it appears from counting genes. Of course one reaches a point where two species live under different enough conditions that comparing their abundances is like comparing apples to oranges. But since expression is highly correlated with evolutionary rates [38] these are probably cases where primary sequence analysis would fail too. More abundance data may shed light on why some branches of the tree are so much harder to resolve than others. Fortunately species most likely to exchange genes horizontally live in similar conditions [23]. This means it will be possible to compare the relative contribution of horizontal and vertical inheritance to the cell when protein abundance data are available from different taxa living in the same environment.

The landscape of genomes is rapidly being filled, and many higher level taxa are now well sampled. Despite this, there is no consensus on the TOL and many are ready to abandon the notion that we will ever reconstruct it. There is still plenty of data that needs to be generated to elucidate the history of cells. More information on protein abundance will shed light on the true revolutions in the history of cells and help prioritize conflicting signals in the genetic material. Protein and cellular structure will help us polarize the major events in evolution. It seems to us that genomes simply are not enough to study genomics. Of course it would be naïve to expect that some new data source will be a magic bullet that will resolve the TOC. Instead we must realize each data source has its shortcomings, many of which cannot be illuminated except in the context of other data. It is not just that we need more data, we need more details. Automated methods fall prey to numerous confounding factors but can still be highly informative. They must always be supplemented by experts whose intuitions have been tempered by careful examination of details from multiple data sources. Therefore the best way to move forward is to take sequence data off center stage and supplement it with these other data sources.

**Figure 2:**
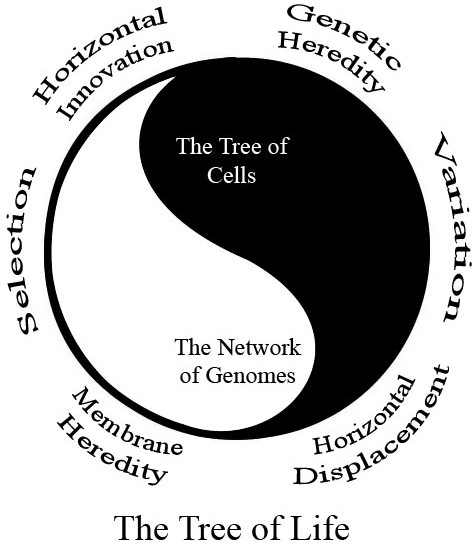
**The yin and yang of evolution**. Several key dualities in evolution are better understood when they are viewed as complements to each other under the framework of the classic symbol of yin and yang. Common descent is the prerequisite for HGT, but horizontal innovation shapes the pattern of descent. Inheritance of genetic material is often web-like, but membrane heredity is tree-like. Both polarities of each of these dualities exist because of the other. The existence of darkness does not invalidate the existence of light, just as the prevalence of HGT does not invalidate the TOC.

This view can be summarized by several dualities, best exemplified by the classic symbol of yin and yang (Figure 2). The basic lesson of this symbol is that one finds the darkest point in the center of the light, and the brightest light in the center of the darkness. It is only through understanding the interplay between the light and dark that one gains insight into their true nature. Neither can exist without the other. A new understanding of evolution comes from the study of the interplay between a series of dualities. It is now clear that there is large distinction between heredity of genetic material and of membranes. There is clearly a duality in Darwin's theory of descent with modification; the history of variation is well described by a network and the history of selection is well described by a tree. A web of life (WOL) may be more factual than a single TOC, but we argue it is a less accurate depiction of life's history. It is possible to precisely represent the relationship between most extant genetic material on this planet using a network. But without a tree (or time line) of life this undirected graph is mostly functional displacements and shifts in redundancy in our opinion. The "light" in the confounding "darkness" of horizontal transfer must be the TOC. Of course the "darkest" points are in the center of the "light" too. Endosymbiosis is clearly a non-vertical event that has profoundly influenced the structure of the tree of function. Likewise there are many horizontal innovations that were important for shaping the prokaryotic tree of function. We feel the most productive way to move forward is create a duality between the horizontal transfers that shape evolution and those that confound our tree building algorithms. The point is that neither of these extremes invalidates the other; they complement each other. Darwin wrote, "Thus, from the war of Nature, from famine and death, the most exalted object which we are capable of conceiving, namely, the production of the higher animals, directly follows." His understanding that death comes from life, and life comes from death fits perfectly with the symbol of yin and yang (the link between the quote and symbol of yin and yang was noted in [44]).

The increase in the size of the functional tree argued here may not be enough to persuade everyone saving the TOL is possible. Some prefer to be cautious and label this the "central trend in the forest of life" [22]. The fact that a large chunk of universal cellular function has remained conserved and its sequence behaves in a mostly tree-like manner after billions of years makes the reconstruction of the TOC seem possible despite the issues of HGT. Resolving and rooting this tree are meaningful problems that are worth pursuing. That is not to say that the tree is resolved simply by taking a consensus or average of universal gene sequences. However, those who rather look at the forest of life need to keep in mind that there are some trees in that forest that are much older and larger than the others. The central tree(s) must be the landmarks used to navigate the rest of the forest. HGT has clearly shaped the prokaryotic world, but if we do not keep in mind the histories of both genomes and cells we will end up lost in the woods.

## Methods

Abundance data were taken from [10,11]. The universal core proteins are defined in [7]. The Enterobacteriaceae genomic core was defined in [19]. The Spirochete genomic core was defined in [20]. All COG annotations were taken from the STRING database [21]. All inconsistency scores were taken from [22].

## Competing interests

The authors declare that they have no competing interests.

## Authors' contributions

REV conceived the study and analyzed the data. PEB assisted in writing the manuscript. All authors read and approved the final manuscript.

## Reviewers' Comments

### Reviewer's report 1

Eric Bapteste, Université Pierre et Marie Curie, UMR CNRS 7138, 75005 Paris, France

This paper, in many respects well-balanced, proposes a strategy to reconstruct a tree of cells, and discusses questions regarding the TOL in ways, that in my opinion, should be significantly improved to be convincing.

The authors begin by acknowledging that genetic evolution is largely reticulated in prokaryotes, but that genetic and cellular evolution should be distinguished, since, importantly, cellular evolution could be accurately described by a tree.

They argue that the tree of cells could be reconstructed by (i) considering the distribution of functions (e.g. the repertoires of functions present in genomes) rather than the repertoires of genes; and by (ii) giving a greater weight to genes that are abundantly expressed in cells (rather than giving similar weigth to all genes) to define the branching pattern of cellular evolution. Genes with a greater abundance, they propose, would be less easy to transfer. In that regard, they introduce a novel (and possibly quite sound) 'complexity hypothesis' based on abundance. In the complexity hypothesis, genes with more interactions are expected to be less transferred, in the 'abundant hypothesis', genes with the most abundant expression are expected to be less transferred.

Whether the evolution of functions is tree-like as the authors repeatedly claim could be tested by reconstructing a phylogenetic network based on the functional content of genomes. The authors should do it in a revised version of the MS and add that analysis and a figure to their paper. This test of the tree-like evolution of functions would improve the paper, since in some parts of their manuscript, the authors mention the problematic possibility that even functions evolution could be to some extent affected by HGT (e.g. p.19). In particular, the extent to which such repertoires of functions could be convergent to adapt to some environments (i.e. animal guts, or hypersaline environments) is probably partly an open question, that could complicate the interpretation of the branching pattern in such trees. Likewise, the authors mention that indels (another type of slowly evolving characters, in their view less affected by HGT) appear on the same points on a tree. It would be tempting as well to see how a phylogenetic network of these data on indels look (e.g. how tree-like the distribution of indels is), and how it matches with the tree of functions.

*Author's response:*

*We are arguing that the tree of function requires a well developed tree of cells to define when lines of cells gained or lost function. Our other work focuses on reconstructing a large portion of the tree of cells, and hints of the tree of function can be seen in it (in preparation). In the tree of functions the source of the function does not matter. The ancestral line did not have the function and the derived group does, which is well described by a branch order on a tree. Whether other branches on the tree have the same function is irrelevant to those cells from a functional point of view unless they are in competition. To really build the tree of functions one would also need to represent the relationships between different functions. We think the history of cells, genomes, and functions are long term goals that cannot begin to be reconstructed in a single figure, but we hope we have argued they are goals worth pursuing still*.

*We agree it would be interesting to map characters such as indels onto networks*.

Gene abundance, especially in extinct cells, may be quite difficult to quantify. The data are currently limited to decide which genes are abundant and which ones are not. Nonetheless, this limitation could be a chance, since it should be possible to test whether these abundant genes evolve vertically or not, by aligning these genes (and corresponding controls) and by searching possible traces of recombination in them, or evidence of inconsistent or odd branchings in their trees. Knowing whether these genes appear to recombine/transfer in proportions comparable with that of non abundant (control) genes would help evaluating the authors' claim that abundantly expressed genes are less affected by LGT. Such an analysis should also be added to a revised version of this paper. Indeed if molecular changes accumulate in these sequences largely due to non vertical processes, at some evolutionary depth, the proposition made by the authors that such genes would better describe vertical evolution than other markers (and thus should be preferred in case of conflict between markers) would simply be wrong.

*Author's response:*

*We have added an analysis of the inconsistency scores for the highly abundant proteins. This data supports our proposal that abundance is a barrier to horizontal transfer*.

The authors' conclusions that the tree of functions (should it be consistent with the data, once reconstructed) is a good proxy of the tree of cells, itself a perfect match of the Tree of Life, is very arguable. The tree of functions, the tree of cells and the Tree of Life can hardly be one same thing. They can hardly be considered isomorphic for a simple reason: they do not have the same explanatory powers, nor the same explanatory scopes. Evolution in general is much more than the evolution of cells, or the evolution of functions (even if these two aspects are very important to understand evolution). The problem is that biological diversity resulting from evolution by far exceeds these two aspects: many evolutionary units (recombined genes, operons, transferred genes, mosaic genomes, consortia, communities, 'acellular' and 'intercellular evolution' mediated by mobile elements such as phages and plasmids) cannot be exactly mapped onto a tree of functions or onto a tree of cells. The evolutionary fates of these objects are partly (and sometimes largely or totally) uncoupled with the ones described by the tree of functions or by the tree of cells. The tree of functions - if it can be reconstructed- would certainly be informative about the evolution of functions; the tree of cells divisions - if it can be reconstructed- will be informative about a part of cellular evolution. However such a tree of cell divisions won't inform us about most of what cannot be considered as mere details in evolution: the lifestyle, adaptation, processes creating and sustaining the genetic diversity, the selection pressures at play and the evolution of species (that is how remarkable groups of organisms emerged (or failed to emerge) from the interplay of evolutionary processes).

*Author's response:*

*You are absolutely right that each of these trees has different explanatory scopes. The tree of functions is certainly not the tree of cells. Our argument is that tree-like thinking is more useful when abstracting beyond the level of genetic material. One would need to combine the tree of cells, tree of functions, and networks of genomes to get the explanatory power of the dreamt TOL. We are not pattern monists, so we have no problem with that*.

*We completely disagree that the trees of cells would not be informative to studying adaptation. It might not provide much explanatory power on its own, but how can one study adaptation without a history of the cell? The tree of cells provides the snapshots of before and after the adaptive process. Mapping any other evolutionary data source back onto the tree of cells makes it more informative. Without the history you cannot say what any of the processes you listed actually changed in a cell. The bottom line is the contribution of these other factors is always on the tree of cells. That is why we argue the tree of cells becomes more important under a pluralistic model*.

This limited explanatory power of such a tree is even clearly demonstrated in this manuscript: the 'cellular core' of four spirochetes is already uninformative about the Spirochetes lifestyles. Using these 'abundant genes' would not allow explaining much of the spirochetes biology, and thus of these species origin (how some become obligate parasites for instance). The duality between the tree of cells and the network of genes, well acknowledged by the authors, seems irreducible, because real and relevant to our understanding of evolution. In other words, while the authors rigthly argue that the genome and the cell should not be confused with one another, they seem to be tempted to approximate the entire biology and the entire evolution by the history of cell divisions. This confusion too should be discouraged.

*Author's response:*

*The tree of function is not just the abundant genes; it would include the losses and gains that define the adaptations to these different environments. We are not arguing cell divisions are all that matter, but rather their history is necessary for a true understanding of these other processes*.

It would certainly be interesting to polarize in time many evolutionary scenarios, but it does not follow that, based on the history of some genes with a slower dynamic and based on some 'frozen' features, we will be able to infer the independent histories of the other genes and of the other organismal properties evolving with a distinct (faster) pace. In that respect, knowing the tree of cell division might not help much in understanding precise gene histories (for instance). A tree of cells will have some useful explanatory power but not as much as a dreamt TOL.

*Author's response:*

*We agree that some genes histories will still not make sense even in the framework of something that resembles the TOL. Our point is that without some branch order for the major prokaryotic groups it becomes difficult to come up a meaningful history for ANY gene. The tree would let us differentiate the slow and fast properties, which would give great insight to evolutionary processes that are either tree-like or network-like*.

The authors' choice to keep using the phrase 'tree of life' when referring to the pattern of common descent even if it is more of a web, because this would somehow annoy creationists, is in my opinion not a good idea. I do not think creationists should dictate us any of our scientific agenda, or influence our wording, as they have no scientific competence to evaluate evolutionary studies. When phenomena are not tree-like, we should not call them a tree. When they are tree-like and are supported by several lines of independent evidence, we should call them the 'most corroborated evolutionary tree' or the 'best evolutionary tree' but not the tree of life, because maybe features did not evolve in a tree like fashion, and thus cannot be reduced to that scheme to be fully understood.

*Author's response:*

*If this was our only reason for this title you would be absolutely correct. We have presented many reasons besides this why we think 'tree of life' is a worthy title for the combination of the histories of cells and genomes. It is not about annoying creationists, which comes quite naturally to us. We agree that they should not dictate our agenda, but clearly they have already shaped our wording. The point is 150 years ago the phrase 'tree of life' invoked a vision of a talking snake and a magic apple. Now it is a story that involves genomes, viruses, and algorithms. The meaning of the 'tree of life' will continue to evolve, but it will continue to provide an explanation of where life came from. We don't think any other title could ever have quite the same aesthetic value, but beauty is in the eye of the beholder*.

There is then a cost to do as if the Tree of Life existed (but not testing this scientific hypothesis): it reifies parts of the tree, like the nodes and the branches. Lawrence and Rechtless have masterfully shown that nodes, when conceived as points of speciations, are not 'real'. When prokaryotic species do not evolve by a series of dichotomies, it is a delusion to impose a dichotomy to describe a speciation.

*Author's response:*

*Lawrence and Retchless *[45]* have demonstrated that nodes are fuzzy in terms of genetic material due to varying levels of recombination during the divergence process. If we consider a tree of functions then the nodes are real. Consider an ancestral state that lacks a function. The function is gained (through HGT or innovation). There is now a derived and ancestral node that can be described on a tree, but the history of their genetic material is no longer so well behaved. Their may be a fuzzy functional intermediate, but that would not be a stable state due to selective pressures. Again trees appear a better data structure if we abstract past the genome*.

The root poses a comparable issue. The authors keep referring to LUCA, as if there were one one last universal common ancestor of all life that was a cell. The literature on early life is, to say the least, divided about this notion. Invoking LUCA to prove that there is a tree, and the tree to prove that there is a LUCA, without any principled way (or any test) to refute that there is a LUCA or that there is a tree is unfortunately a circular argument. That all cells share a given gene/function does not mean that all cells evolved vertically. If LUCA ever existed (which I doubt, and most importantly which in my view could explain more than a part of early evolution), how big was its pangenome? What kind of mobile elements drove its evolution? We need to make sure that a prioris about the tree and about LUCA being real do not already bias our conclusions, if these a prioris cannot be tested. Otherwise, we might reinforce our habits of tree-thinkers, but not necessarily our knowledge of evolution per se.

*Author's response:*

*The issue of LUCA is certainly muddled in the literature. We direct the readers to a recent empirical argument that there must have been a LUCA, even if it was a community *[46]. *You are right in pointing out we are biased in our view of LUCA. Our other work has led us to support Cavalier-Smith's assertion the Chloroflexi are the earliest branching extant group. Therefore, we assume LUCA had a relatively small pan genome. If one accepts the canonical rooting between the Archaea and Bacteria the idea of a large pan genomed LUCA is certainly more appealing because of the large differences between the prokaryotic superkingdoms. If LUCA was a large pan genome our focus should be on what genes could NOT have been in that community and must be younger than LUCA*.

In that regards, I have a few detailed suggestions where some simplifications could maybe be corrected in the text.

p.3: The authors wrote: If every daughter cell's membrane kept track of who its parent was, reconstructing evolution would be a trivial task. I feel that this is a bit misleading, it would inform us on a part of evolution: If every daughter cell's membrane kept track of who its parent was, reconstructing evolution **of cell divisions **would be a trivial task. It would not tell us anything on how species taxa, and genes, and phages, and communities, etc. evolved.

*Author's response:*

*A more precise statement, which we have adopted*.

p.3: It is very arguable that even genomic evolution makes little sense without the light of cellular evolution. There is certainly lots of knowledge to be gained from metagenomic analyses, from the study of mobile elements, from the study of gene evolution, lots of patterns and processes to explain, even without the light of cellular evolution. This is not to say that we would not benefit from that particular light. But this light will mostly make a 'genealogical' sense on evolution, and evolution is more than genealogy.

*Author's response:*

*Studying evolution without genealogy makes little sense to us. These are all important processes, and we certainly can learn a lot about them without the TOC. However, we argue the TOC gives a deeper understanding of each of them. Without considering genealogy in metagenomics one basically has a laundry list of genes, and it might not even be clear which of them are from the same cell. If one has knowledge of how the cells in the community are related, they can reconstruct the history of the mobile elements and examine what their impact on the community was. Likewise studying gene evolution without genealogy seems limiting, as the impact the gene has is ultimately on the fitness of the cell. The genealogy is necessary to integrate these processes into a bigger picture and to see what they actually changed*.

p.4: The TOL does not become even more important under a pluralistic approach, quite the opposite: it is regionalized under a pluralistic approach, as no single model can explain everything about evolution under that perspective. Finding the tree of cells for instance remains an important and ambitious goal, but not the alpha and omega of evolutionary research. The importance of the TOL thus decreases while the importance of additional interesting evolutionary questions increases.

*Author's response:*

*We have changed this to TOC in the text. We are arguing building it is the alpha, but not the omega. The fact that the TOC will be used to formulate more questions than initially expected makes it more important*.

p.5. It seems to me that proteins abundancy and core cellular features might be a basal make-up of cellular lineages on which further adaptations are adjusted. If there is some ratchet, abundant proteins can not be easily gotten rid of, but that does not mean that most of the evolutionary dynamic concerns these proteins and their coding genes.

*Author's response:*

*Evolution is certainly not just about the abundant genes. We have included abundance data to add a dimension beyond the genome to study the big picture in evolution*.

p.5. Comparing how many of the same functions two cells are doing at the same time is a good measure of similarity, but is it a good proxy of the genealogy ? This depends on the amount of convergence and selective pressures on functions induced by the environment. Is not it possible that bacteria of the gut microbiomes (or of a salty environment) will perform the same functions at the same time even if they are not directly related ?

*Author's response:*

*This is certainly a vital question to answer. Abundance data from similar environments will allow us to test this in the future, but for now we are left to speculate*.

p. 6. For a detailed argument that a TOL is not as meaningful as claimed in [13], when there is a large incongruence between individual gene trees, see Bapteste et al. Biol Direct. 2009 Sep 29;4:34. Prokaryotic evolution and the tree of life are two different things.

*Author's response:*

*We lean towards your arguments in this case unless one can deal with the incongruence in a non-arbitrarily manner as we have proposed here*.

p.11: I absolutely disagree with the following statement: Trees have many shortcomings for representing prokaryotic evolution, but any data structure that lacks temporality is even worse. Reconstructing a wrong tree (imposing an irrelevant structure to the data because of our a prioris) is the worst thing one can do. We can learn a lot from unrooted gene trees, on which by definition there is no temporality.

*Author's response:*

*We mean that we need the benefits of the trees we have listed, while trying to accommodate the shortcomings of that representation. We are not for forcing data to fit the tree. We are saying the data that does not fit the tree can only be noticed and studied once you have the tree. There are certainly other representations that are useful, but the TOC is necessary for weaving them into a coherent story*.

p. 18 and p. 19: When commenting on dualities, the text becomes pretty metaphysical in places. I do not see why 'the' light must be 'the TOL': a light can be a tree of cells, another light a tree of functions, and so on. Why do you need only one light to explore the darkness of evolution anyway, while so many processes are occuring, creating a diversity of phenomena that calls for more than one explanation ?

*Author's response:*

*We think a discussion of the metaphor used to represent the process of life should have a healthy dose of metaphysics. The nice thing about yin and yang is that you can switch them and it still tells the same story. We have chosen light because in this case the answer the TOL provides (if it exists) is more directly readable than the WOL. There should be multiple lights, but we cannot see how anything could be more useful to understanding the history of genes than understanding the history of cells. These are the two primary replicative processes in evolution. The assumption they were the same process is the source of the problem. As we begin to separate them we must keep them connected. Therefore we feel the yin and yang is fitting. One could argue the confusion caused by forcing data to fit a tree is the darkness and the light is the realization of processes like HGT. The point is that HGT and the TOC are inseparable and cannot exist without the other, regardless of how we label each one*.

A thinking about evolution in terms of yin and yang is possibly not entailed by the quote on (I believe) Darwin's malthusianism. I doubt that historians of sciences and/or philosophers of sciences would be convinced that this is a proper use of that particular quote. I do not think it is needed in the paper.

*Author's response:*

*We probably did go a bit too far in our use of this quote. We have softened the implications, but have kept the quote. We see the yin and yang in that quote, as well as in the data, regardless of what Darwin was thinking when he wrote it*.

p.18: I disagree with this sentence: A web of life may be more factual than a single TOL, but we argue it is a less accurate depiction of life's history.. The authors possibly have in mind a fairly simple web of shared genes. But even these graphs can be further studied to gain knowledge on history. Dagan and Martin for instance have shown how such networks can be exploited to learn about life's history. And what about phylogenetic networks for taxa with a limited amount of HGT: are they worse than a tree to describe life's history? To me this kind of claim is counterproductive, as it fails to acknowledge that it might just be time to change our habits and our thinking about how evolution should be described.

*Author's response:*

*We are making a similar point to the one you made above about explanatory power. The WOL does explain as much as the dreamt TOL. Therefore we would be settling for too little if we thought it was enough*.

I also would like to make some further precisions:

p.2. The conclusion of Doolittle and Bapteste is not so much that the inability to build the tree is the problem, rather it is forcing the data into a tree that needs to be questionned, and in a pluralistic framework, avoided, since this model does not allow a precise description of the evolutionary processes.

*Author's response:*

*Changed*.

p.17: The authors write that it is not clear what level of genome conservation between strains of prokaryotes would be satisfying enough to consider them evolving in a tree-like manner. It is true that two strains of the same species may have relatively few genes in common, but we have argued above this is probably an exaggeration of functional distance. Even if the second sentence might be correct, its association with the first one suggest that the authors tend to overlook the importance of recombination in prokaryotic genomes, a major process that is not tree-like. This non-tree like phenomenon can in part be masked by zooming out at a higher taxonomical level, still the real processes responsible for evolution are not tree-like. In that respect, a tree of cells or a tree of functions will fall short in explaining major evolutionary processes at play on genomes.

*Author's response:*

*We are trying to emphasize the many tree-like patterns that could be masked by recombination of genetic material. Certainly a network is needed to understand the history of the genome. Our key point is that does not mean a network necessarily describes the evolution of cellular function better even in the presence of recombination*.

p.17: Just like genomes simply are not enough to study genomics, cells (or functions) are not enough to study evolution: you need to include phages, plasmids, etc.

*Author's response:*

*True, but in reality phages and plasmids only really affect evolution when they enter cells*.

p.19: The duality that the authors propose between HGT that shape evolution and HGT that confound our tree building algorithms seems a distinction between good and bad HGTs. This distinction (if it can be achieved, how ?) could help them building an evolutionary tree, but it would not make the processes of evolution and prokaryotic genetic evolution more tree-like in nature. Both good and bad HGTs are non strictly vertical processes.

*Author's response:*

*This distinction can only be made by having a hypothetical ancestral genome before the transfer occurred, which is not a trivial task is given the amount of transfers that have occurred. If the function is novel to the recipient cells lets call it a good HGT. In both cases there is non-tree like evolution, but we argue that bad HGTs are just red herrings leading us away from the tree that does exist. Our point is that HGTs are not strictly non vertical from a functional perspective*.

Overall, in many places of the MS the authors could advantageously replace TOL by Tree of Cells, which would address (simply) most of my concerns.

*Author's response:*

*Calling the TOL a universal sequence tree in much of the current literature would have addressed many of our concerns as well, but your arguments have convinced us TOC is more precise in several places. We think a compromise is to use the term TOL to refer to the combination of the network of genomes and tree of cells. It would not be entirely a tree, but it would have nearly same explanatory power as the original TOL hypothesis. We hope this work adds precision to these terms instead of just muddying the waters*.

To sum up, I feel that the current title of the manuscript is misleading, unnecessarily dramatic, and should be modified.

*The title is meant to be dramatic. We have explained what mean by 'lost in the woods' a little better in the introduction. Now that we have changed TOL to TOC in many places we explain the title as rescuing the explanatory power of the TOL by remembering the WOL needs to be grounded in the TOC. We feel the need to be dramatic because many appear ready to abandon the TOC because it is confused with the TOL*.

What this MS proposes is how a tree of cellular functions, equated with the tree of cells, could possibly be reconstructed by taking into account additional (novel) sources of data (such as the functional repertoire of genomes and the abundance of expressed genes in the cells) rather than by focusing on the mere gene content of genomes, and by giving comparable weights to the phylogenetic signal(s) of each individual gene.

The Tree of Life and the tree of cells are however two different things: in particular they do not offer similar explanations of evolution. The tree of cells is by definition more limited in its scope than the legendary Tree of Life. It is then important to stress that the tree that could be saved if the author's proposition hold is (and that is already quite good) either the tree of functions or the tree of cells.

The title also suggests that evolutionary biologists would be lost without this one tree. I think this claim is unduly pessimistic, and stems from our acquired habits to explain evolution with a tree model. Evolutionary biologists won't be lost without the tree of life: they will be challenged. They will need to reconsider their practices, their goals, and their explanatory toolkits to make sense of an evolution that is not just tree-like.

There are lots of fascinating researches to be done to learn about the evolutionary processes and mechanisms, that do not require the inference of a unique tree of life, i.e. to harvest the phylogenetic forest of unrooted trees (see Lapointe et al. Trends in Micro, in press), or to exploit genome networks (see Dagan and Martin's works; Fani, Fondi et al.'s works, Lima-Mendez, Leplae et al.'s or Halary et al.'s works). Our explanations will be different, but evolutionary biologists won't be out of job or hopeless. Such a possibility could/should have been explored more by the authors, as they reckon that it is not clear what our expectation of prokaryote should be. Precisely, clarifying this expectation, with the least possible a prioris, is an exciting prospect for evolutionary science.

*Author's response:*

*Processes and mechanisms are certainly important, but evolution is about history to us. We only care about the mechanisms and processes because they caused the history. You cannot understand the mechanisms or processes without the history. We certainly believe that networks have a lot to teach us, but they only are meaningful when grounded in a TOC. Therefore the TOC becomes more important as we try to understand the processes that do not fit into that scheme*.

This is why I finally beg all authors who might be tempted to send me some more papers to review on themes such as 'rescuing the TOL' or 'saving the TOL' in a near future not to: I have definitely said all I had to say on that issue for a little while, and it is time for me to move on more exciting research topics ྶ.

*Author's response:*

*We sincerely thank you for one last round on this subject. Unfortunately for you, your insightful review of this manuscript will probably make others want to continue this discussion with you. But we understand the need to move on from this topic*.

### Additional specific comments

#### Further questions

p.8: Do large pangenomes have larger repertoires of functions ? If so, won't that affect the reconstruction of a tree of functions ?

*Author's response:*

*A large pangenome may have a large amount of functional redundancy. This will not be a problem if one has a good functional outgroup, but that requires a well defined TOC*.

p. 9: Why should we assume that COGs that are the most widely distributed in extant taxa are the most ancient ones? Why can not they be highly transferred ?

*Author's response:*

*You are right that some of these could be the result of frequent transfers. To the best of our knowledge there is no case of a young protein being transferred to the majority of a superkingdom, but there are many proteins clearly in the ancestor of a superkingdom that have been retained. Therefore in general the most widely distributed proteins are the oldest. It seems we would need the tree of cells, tree of functions, and network of genomes to be certain though, so for now this is a reasonable estimate*.

p. 19: The fact that a large chunk of universal cellular function has remained conserved and its sequence behaves in a mostly tree-like manner after billions of years earns it the title of TOL in our opinion. How is this a fact ? How has this been tested ?

*Author's response:*

*We have changed this one to TOCs to soften it. We are calling the 5% of the 'abundome' represented by the universal proteins a large chunk, which is certainly arguable. However, this number increase if one considers the functional content of the last common ancestor of each superkingdom instead. The nearly universal trees in *[22]* have a high level of consistency. Therefore, we think this statement is justified in its current form*.

#### Unclear sentences

p.1. 'results': What do you mean by ***proper ***selective context ?

*Author's response:*

*Differentiating between whether it is a good or bad horizontal transfer*.

p.2. Why should the woods be 'woods of ***neutral ***evolution' ?

*Author's response:*

*Because we believe most of the noise coming from HGT are actually just displacements*.

p.5. What do you mean by: The downside of abundance is it is dynamic, while genomes are static. ? What time-line/evolutionary scale do you have in mind ? At the TOL level, genomes are very dynamic.

*Author's response:*

*It is true on evolutionary time scales genomes are dynamic. We mean within a single cell*.

p. 12: This entire section: The disagreement between these phylogenies is not in terms of how to define the major taxa but rather in the proper way to polarize the data, especially the indels (insertion deletions) which we have discussed [34]. However, the distribution of these traits themselves implies specific taxa evolved before another, regardless of the direction of each polarization. For example, there is a large insert in HSP70 (heat shock protein 70) that is present across the Gram-negative bacteria, but absent in the Gram-positives. One form of the protein must have predated the other. There is no reason to assume all the informative indels were fixed early in evolution, and one would be very hard pressed to draw a detailed scenario of transfers that explains their distribution better than a more timeline like structure. is unclear, and should be somehow rewritten. If this is a philosophical point (rather than an empirical comment on the data distribution), I would say that the best explanations are not necessarily the ones that match a tree, these latter are only the simplest explanations. When irrelevant, they do not help much.

*Author's response:*

*This is an empirical comment. We arguing if HGT was truly so rampant as to annihilate any trace of the TOC it should not be possible to find independent traits that support these phylogenies. We have rewritten it to try to make our point clearer. We see no need to invoke a more complicated explanation if the simple one works*.

p.13: Abundance data may make it possible to quantify what Simpson coined "quantum evolution" when referring to the metazoan fossil record [35] on a molecular level in prokaryotes. This sentence needs to be developped or better explained (as it is I do not recognize Simpson's theory - that gives a main role to the environmental selection in quantum evolution- if I recall correctly, as a particularly valid analogy here).

*Author's response:*

*We have inherited our use of this term from Cavalier-Smith. To be precise we mean events where there is a domino effect across numerous proteins that results in rapid evolution. We are arguing that if some major change in abundance was tolerated by rapidly shifting the abundance of other proteins it would very difficult to resolve with sequence data regardless of HGT*, p. 16: This highly finctional sentence makes no sense whatsoever to me: We think the TOL crisis would be worse if it was the "tree of 99%", as it would be quite difficult to explain the phenotypic differences between humans and *E. coli*. It is remarkable any genes are conserved since LUCA, and therefore the TOL still rings true to us.

*Author's response:*

We are saying there tree of 1% argument makes no sense without a null hypothesis. This purely fictional sentence is an example of another tree we could be dealing with that would cause a different set of problems. Put another way, what% did the community expect to be conserved before genomic sequences were available and why?

#### Typos

p. 2: Bapteste is spelled strangely.

p.17: But since expression is highly correlated without evolutionary rates [38] Do you mean 'with' not without, don't you ?

p.19, first line: a word is missing before 'has'.

*Author's response:*

*We have corrected all of these*.

### Reviewer's report 2

*Arcady Mushegian, Department of Binformatics, Stowers Institute for Medical Research, Kansas City, Missouri, USA*.

I have read the manuscript by Valas and Bourne with considerable interest, wholeheartedly agreeing with several ideas in it and disagreeing with some. The best home for this study is probably in the *Opinion *category within *Biology Direct *- this is not really a research paper.

*Author's response:*

*We feel the paper is both research and opinion, and hopefully it will fit fine in either category*.

There are two main themes, one of which is more of a research proposal, the other more of a philosophy-of-science talking point. The research proposal is essentially to enhance the utility of genic traits by assigning weights to them - the weights which, directly or indirectly, estimate relative contribution of each gene to the phenotype; if I understand the proposal correctly, the significance of the phylogenetics signal can therefore be reordered by the "functional rank" of the sequence from which this signal was obtained. I think this is a good proposal, and Adami/Wilke and Koonin's groups, among others, have already said a lot about gene "relative importance"; important technical details of all that have not been worked out, however. The more methodological question, of what to make out of the purported lack of tree-like signal, or of the Doolittle and Bapteste's "pattern pluralism" and other related proposals, is also of interest, and my intuition runs close to the author's, but I still think that he is led astray by the setups of the problem in the literature.

In more detail, much of the "conceptual" literature on the HGT is hand waving about "rampant", "massive" etc. aspects of horizontal transfer. This usually refers to the large number of events observed in a particular dataset, but generally fails to acknowledge that this high *number *of events usually accounts for a small *proportion *of the genes in the dataset and correspondingly relatively *low average ratio *of horizontal to vertical branches in the trees. (Ninety-nine percent of the trees, for example, may show some evidence of HGT, but in the vast majority of these trees, there may be just one or very few HGT events, and so on; see, e.g., Pubmed 19077245, 18062816 and 15799709). Thus, instead of talking about the applicability of the TOL "metaphor", perhaps we should be talking about TOL quantitative model, the alternatives to it, and which model or mixed model is best compatible with the data.

*Author's response:*

*We agree with your sentiment towards these results. We are all for a mixed model, but it needs to be a true duality where cells and genomes are treated as such, instead of just a reticulated network of genomes*.

The author states about Doolittle and Bapteste's proposal: "A key point of their work is that any data can be forced to fit a tree, even if that representation of the data makes no sense" - in fact, this has been known for a long time; the same can be said about any representation of the evolutionary process (e.g., alignment algorithm will align even unrelated sequences, and network algorithm will build a network even on a hierarchical set of OTUs); and finally, so what?

*Author's response:*

*We have changed this sentence to one suggested by Eric Bapteste in his review. The other two examples you bring up are valid. It is easy to forget the results of high powered computing tools we have still are prone to 'garbage in garbage out'. Some alignments are probably forced to fit, but the authors are saying ALL universal trees are forced to fit. It took their persuasive argument to demonstrate how forced many of the genome trees are. It is the scale of the problem that makes their work worthwhile*.

In other words, the author should stop fighting the windmills: the goal of phylogenetics should not be to build a tree, nor to build any graph with another kind of predetermined topology, but to improve our understanding of which evolutionary events actually happened and led to the observed data. I think this accommodates "pattern pluralism" naturally.

*Author's response:*

*We agree, but one must have some data structure in mind when designing algorithms and strategies to reconstruct these histories. We feel trees capture the history of the events better, and that is needed to supplement the networks to get anywhere*.

I would advise to the author to get all this out of the way early and to focus on the relatively independent proposal of including protein abundance and other information (such as perhaps correlated essentiality and degree of conservation) into the judgement of importance or relevance of any particular tree topology for phylogenetics. I would like to see the discussion of several points in more detail.

*Author's response:*

*We'd rather let the reader see our wild speculation and eastern symbolism after a little well grounded research*.

1. "A cumulative plot of genomic and cellular abundance reveals that at every level genomic abundance underestimates cellular abundance" - so what does this tell us about phylogeny? Also, the datasets that are available to us are full of parasitic microorganisms whose genomes may be experiencing net gene loss, which contributes to the reduction of the "genomic abundance" of almost all categories of genes. Would the picture change if we focus on free-living/saprotrophic organisms?

*Author's response:*

*This implies that when a large group of trees is in agreement about phylogeny that forest is a large portion of those cells. That makes the phylogeny more historically real to us. It would certainly be interesting to focus on abundance in parasites and their free living relatives. We assume that most of genes they retain would be highly abundant proteins in free living cells, and they mostly streamline what is usually necessary to power that core. That would be consistent with higher levels of conservation when measured by protein versus genomic abundance in the spirochete data set, but more data here would certainly be informative*.

2. The authors want to rescue the tree by bringing in the functional importance/protein abundance (phenotype), as discussed before. In this case, would not the change of function be equivalent to a HGT, and would this be less or more often than a true genetic HGT?

*Author's response:*

*Functional changes are never neutral, and we argue HGTs are neutral most of the time so they are not equivalent. It is hard to speculate on the frequency of such events because so there are so many ORFans in sequenced genomes, and so many proteins beyond that which have not been functionally characterized. If that portion of genomic space represents nuanced novel functions they could be more frequent than HGT. We think this is exactly the sort of question that requires both a tree and a network to answer properly*.

### Reviewer's report 3

Celine Brochier, Laboratoire de Chimie Bactérienne, CNRS-Aix Marseille Université, 31 Chemin Joseph Aiguier, 13402 Marseille Cedex 20, France

In this paper Valas and Bourne propose an original approach to reconstruct the tree of life.

To my point of view this contribution is more an opinion than an experimental paper. This is at odds with the organisation of the paper that includes a large "results" section (11 pages), whereas the real experimental part of the work is represented by a single figure and one paragraph (1 page). By contrast, the discussion" section is rather short (3 pages). I think it would be more appropriate to combine the results and the discussion sections into a single section, with subdivisions corresponding to the different points that are discussed. Finally, I think it is important to clearly classify this manuscript as an opinion and not as an experimental paper.

*Author's response:*

*We have combined the results and discussion as suggested. We agree that this paper is not a traditional research paper, but we still feels it belongs in that category as it is a combination of novel research, opinion, and review*.

The contribution of Valas and Bourne comes within the scope of the hot debate around the Tree Of Life (TOL). Indeed, based on genomic data the suitability of tree-like structures to represent the evolutionary history of all organisms has been highly debated [4,8,47]. The two main arguments are that in prokaryotes (1) the evolutionary history of genes is different from the evolutionary history of organisms because of horizontal gene transfers (HGT) [4], and (2) HGT may be so frequent that a substantial part of the genes in a genome have been affected by HGT. Then, jumping from genomes to organisms (perhaps because we have entered in a "*too genomic-centric*" area, as stated by the authors), this has led to the conclusion that, at least for prokaryotes, a tree-like structure does not reflect the evolution of genomes, which will be better represented by a network (NOG, Network Of Genes). However, if nobody can deny that HGT have played an important role in evolution (and not only in prokaryotes), it is also undisputable that cell division in prokaryotes occurs by the division of a mother cell in two daughter cells. It is therefore theoretically possible to trace-back the history of cell lineages and to represent it with a tree-like structure, the TOL. However, the TOL and the NOG are often confounded, maybe because genes are the only informational entities that are transmitted from one generation to another, whereas TOL and NOG represent two different things that are equally interesting and highly complementary to understand the evolution of living organisms [5,14]. The authors state these points well by writing: "*All of the issues the community is currently having with the TOL hypothesis stem from the simple fact that genomes are not perfect representation of membrane history*. [...] *even genomic evolution makes little sense without the light of cellular evolution*".

The challenge is now to reconstruct the TOL in a NOG context. Classical approaches consist to identify (and to analyse) the sets of genes that may be used to reconstruct the different parts of TOL. This step is important because it appears a utopia to think that it is possible to fully resolve the TOL (from the root to the leaves) based on the analysis of a few universal genes. This would be the tree of 1%. In fact, it would be cleaver to divide the problem by looking at the set of genes suitable to reconstruct different parts of the TOL. For example, the set of genes suitable to resolve the phylogeny of animals will probably be different to the set of genes that may be used to trace back the relationships within Methanococcales (Archaea). This is well known by botanists and zoologists who used different sets of characters for different levels of their classifications. Ideally, the TOL should be a synthetic drawing showing the relationships between organisms (not species, which are artificial entities, or genomes) by combining the results obtained by the phylogenetic analyses of different sets of genes. In this case, the TOL will not be the tree of 1% but the tree of dozens of percents, each gene contributing to resolve some parts of the TOL.

Here the authors propose a radically different approach based on the vertical inheritance of functions rather than on the vertical inheritance of genetic material. The approach is based on the assumption that all genes do not contribute equally to the cell: some are more important than others. The authors underline that the gene contribution to the cell should be an important criterion to take into account when reconstructing the TOL. As the authors point out there are different metrics to measure the gene contribution to a cell: "*essentiality, abundance of proteins, number of transcripts, portion of total weight, etc*".

1) My first question is how to organize these factors in a hierarchy, i.e. which criterion is the more suitable to represent protein importance in a cell? And what do these factors exactly represent from an evolutionary point of view? Is it possible to develop evolutionary models for such data (that are mainly quantitative and not discrete characters)? Unfortunately the authors do not propose methodological approaches to analyse such data. I think this is important to discuss about their suitability to reconstruct the TOL.

Among these factors, the authors chose to study the abundance of proteins in cells (the "*adundome*"). Based on recently published data on the abundance of proteins present in the cytoplasm of *Escherichia coli *cells (GammaProteobacteria) and of the complete proteome of *Leptospira interrogans *(Spirochaetes), the authors argue that "*abundance is a good proxy for evolutionary importance because there is a correlation between the abundance of a protein and the energy the cell invests into producing it*".

*Author's response:*

*We do not have precise answers for these questions, but they are certainly going to be important to answer. Our demonstration that abundance is a barrier to transfer supports the notion it is evolutionary important. But there are abundant genes that have been transferred. It would be naïve to say those are less important. It might not be possible to precisely quantify how important each gene is to the cell, but we have demonstrated that in general the important genes evolve in a more tree-like manner. We are hopeful it will be possible to develop evolutionary models for the evolution of "abundomes", but we doubt they will behave well enough to resolve the TOL or TOC on their own. Rather we think they will be tools to help us understand how the TOC was shaped. In either case it would certainly be premature to begin developing these methods from the two datasets currently available as they are not directly comparable. A sampling of many strains of E. coli seems like a good place to start addressing these ideas*.

2) This raises my second question: what does the "*evolutionary importance*" of a protein mean from an evolutionary point of view? The word "*importance*" is a subjective and indefinite criterion. The abundance is one side of the importance, essentiality is another. Indeed, a protein may be important even if it is not abundant in a cell (e.g. transcription regulators). The authors should discuss more this point.

*Author's response:*

*Importance is certainly a subjective term. We are not arguing that abundance is a perfect representation of importance but it is seems to be a straightforward and objective measure. We argue that as we measure the importance of function in some meaningful way the importance of HGT will begin to shrink, and the vertical component will grow in size*.

3) More problematic, and this is partially raised by the authors, the abundance of a protein is a dynamic parameter that may vary across cells depending for example of their lifestyle. More importantly, for a given cell the relative abundance of its proteins may vary in time, depending for example on the developmental state reached (e.g. cells in exponential growth or in stationary phase, etc) or environmental conditions. How to take this difficulty into account?

*Author's response:*

*There are certainly going to be many difficulties in using abundance data. We argue the proteins that remain abundant under a variety of conditions are probably the most important, but surely there will be many interesting caveats to discover as more data become available. Again, we are not suggesting using this data to build phylogenetic trees, but rather as a tool to better understand the ones created from other data sources*.

4) I think the large paragraph on indels and the timing of appearance of prokaryotic phyla should be removed because it is beyond the scope of the paper, and I am afraid that the reader will loose grasp on the logical succession of ideas. Same remark for the paragraph dealing with protein structures. On the contrary, I think the authors should rather focus on their proposal to use "abundome" to reconstruct the TOL and in particular on the methodological aspects.

*Author's response:*

*We are not arguing the "abundome" data can reconstruct the TOC, but we think the indels and quaternary structures can. In some sense we are using abundance data to show the phylogenies created using other data sources are meaningful despite the arguments against the TOL. Most of these arguments are against sequence based methods, so we think it is appropriate to include our other work as a demonstration the TOC is still evolutionary meaningful and can still be resolved*.

5) Finally, I have a few comments regarding assumptions that are made on phylogenetic studies based on gene sequence analyses. The authors say that "*current methods for estimating HGT rely on measuring inconsistencies between sequence trees or looking for unusual compositional features, so there is no way for them to distinguish between innovations and displacements*". I think this statement should be toned down because, in the case of phylogenetic studies, most of the time a careful examination of trees allows discriminating between gene acquisition and gene replacement. This is for example the case for aminoacyl-tRNA synthetases (that are discussed in the paper), where clear cases of gene replacements can be identified.

*Author's response:*

*The aminoacyl-tRNA synthetases are a special case because the combination of their trees and knowledge of the essentiality of their function implies these are displacements. It is much more difficult to conclude that from the trees alone. It is not impossible to discriminate between these scenarios, but it seems like many do not seem to worry about the difference when looking at forests of phylogenetic trees*.

Very minor points:

I do not understand the sentence "*We argue events like this are far more deleterious to tree reconstruction algorithms than they are to the recipient cells*."

*Author's response:*

*We feel that sequence has persisted as the primary tool to study evolution because of the relative ease algorithms can represent it, as opposed to these other sources of data. Again we are trying to emphasize the difference between displacement and innovation needs to be made by including other data sources*.

The legend of Figure 1 is poorly understandable.

I did not understand the last sentence of the abstract.

*Author's response:*

See our reply to Eric Bapteste

I disagree with the allusions to Darwin in the discussion section "*There is clearly a duality in Darwin's theory of descent with modification; the history of variation is well described by a network and the history of selection is well described by a tree*". First, this sentence is not clear. Second the history of variation may be represented by a tree: for example, the evolutionary history of a gene (irrespective to HGT) may be depicted by a tree and it is possible to indicate on each branch the mutations that occurred, and therefore to follow the history of variation of this gene.

*Author's response:*

*We are speaking in very general terms. Your example is correct, but the variation of organisms is in terms of their entire genomes. The gene cannot really be selected for independently of the rest of the cell and genome, so the variation is relative to them as well. It was assumed for a long time the history of these two processes is the same, and we think it has become time to explicitly separate them*.

## Supplementary Material

Additional file 1 ***E. coli *data Columns A-E of additional file **1** are taken directly from supplemental material of **[10]. The universal core proteins are defined in [7]. The Enterobacteriaceae genomic core was defined in [19]. All COG annotations were taken from the STRING database [21]. All inconsistency scores were taken from [22]. Click here for file

Additional file 2 ***L. interrogans *data**. Columns A-D of additional file 2 are taken directly from supplemental material of [11]. The Spirochete genomic core was defined in [20]. All inconsistency scores were taken from [22]. Click here for file
